# A Mobile App Lifestyle Intervention to Improve Healthy Nutrition in Women Before and During Early Pregnancy: Single-Center Randomized Controlled Trial

**DOI:** 10.2196/15773

**Published:** 2020-05-15

**Authors:** Matthijs R van Dijk, Maria P H Koster, Elsje C Oostingh, Sten P Willemsen, Eric A P Steegers, Régine P M Steegers-Theunissen

**Affiliations:** 1 Department of Obstetrics and Gynaecology Erasmus Medical Center University Medical Center Rotterdam Rotterdam Netherlands; 2 Department of Biostatistics Erasmus Medical Center University Medical Center Rotterdam Rotterdam Netherlands

**Keywords:** mHealth, preconception care, nutrition, pregnancy

## Abstract

**Background:**

Unhealthy nutrition contributes to the worldwide rising prevalence of noncommunicable diseases. As most adverse reproductive outcomes originate during the periconception period, effective interventions targeting this period are needed. Therefore, we developed the lifestyle intervention Smarter Pregnancy to empower women to adapt a healthy diet prior to conception and during early pregnancy and performed a randomized controlled trial.

**Objective:**

The objectives of this trial were to investigate compliance and effectiveness in women using the Smarter Pregnancy program.

**Methods:**

Women aged between 18 and 45 years who were contemplating pregnancy or <13 weeks pregnant and their male partners living in the urban area of Rotterdam, the Netherlands, were eligible for participation. After baseline screening, the intervention group received personal online coaching based on identified inadequate intakes of vegetables, fruits, and folic acid supplements. The sum of these risk factors was used as a dietary risk score (DRS), ranging from 0 (healthy) to 9 (unhealthy). The control group did not receive coaching. We applied an intention-to-treat principle and used a multivariable linear regression model to evaluate the change in DRS after 24 weeks. Compliance was defined as the percentage of women who completed the screening questionnaire at 24 weeks.

**Results:**

Of women recruited, 81.2% (177/218) completed the program (intervention: 91/218, 83.5%; control: 86/218, 78.9%; *P*=.95). After 24 weeks, the reduction in DRS of women in the intervention group was significantly larger than in the control group (β=.75, 95% CI 0.18-1.34). This reduction was mainly due to increased vegetable intake (β=.55, 95% CI 0.25-0.86).

**Conclusions:**

The high compliance and the larger improvements in nutritional behaviors, especially vegetable intake, in women in the intervention group emphasizes the effectiveness of empowering women by using the lifestyle change intervention Smarter Pregnancy.

**Trial Registration:**

Netherlands Trial Register: NL3927; https://www.trialregister.nl/trial/3927

**International Registered Report Identifier (IRRID):**

RR2-10.1186/s12884-017-1228-5

## Introduction

Unhealthy nutrition contributes to the development of noncommunicable diseases (NCDs) such as obesity, diabetes, and cardiovascular and metabolic disease [1-5]. In recent decades, the worldwide prevalence of NCDs and corresponding mortality rates have increased rapidly [6]. Vitamin deficiencies and high caloric intake combined with inadequate physical exercise are key risk factors for metabolic and endocrine derangements that contribute to obesity and a wide spectrum of NCDs [7-9]. These risk factors are also highly prevalent in women and men during the reproductive phase of their life, with significant consequences for fertility, growth, and development of the offspring [10-17]. Moreover, unhealthy nutrition and lifestyle also confer increased transgenerational risks for offspring in developing NCDs in later life [2,5,18-20].

There is increasing evidence for a need for effective interventions to improve nutrition and other modifiable risk factors in women who are contemplating pregnancy, particularly in the periconception period (ie, the period 14 weeks prior to conception up to 10 weeks after conception) [19,21]. As most adverse reproductive and pregnancy outcomes originate during this period, it is considered the earliest window of opportunity for interventions. However, since the periconception period is often neglected in regular health care, with specific periconception care rarely implemented, the prevalence of these modifiable risk factors still remains very high in the population of reproductive age [10,22].

In order to translate the scientific evidence currently available into accessible periconception care, various barriers need to be overcome. These barriers include the lack of intrinsic motivation for changing lifestyle in the target population; low levels of awareness; and a lack of clarity regarding responsibility, organization, and costs [23-26]. One way of overcoming some of these barriers is to make use of recent developments in electronic health (eHealth). These include using the broad range of functions available on mobile phones and handheld devices, with or without internet access, also known as mobile health (mHealth) [27,28]. Indeed, the global use of smartphones has opened new doors for health care delivery: new and innovative approaches in the fields of preventive and personalized medicine can provide patients with both general information and individualized content [27,29,30]. In 2011, we launched a lifestyle change intervention called Smarter Pregnancy that aims to empower women and men to adopt healthy nutrition and lifestyle behaviors before and during pregnancy. This program is based on models for behavior change and existing evidence regarding the impact of nutrition and lifestyle on fertility and maternal pregnancy and birth outcomes and provides individual coaching on five major risk factors: inadequate vegetable, fruit, and folic acid supplement intake and smoking and alcohol consumption [10,12,13,28,31-34]. Since an inadequate daily intake of fruit and vegetables are the most prevalent risk factors for unhealthy nutrition and users appreciate interventions that are as simple as possible, we hypothesized that stimulating the intakes of these healthy food groups would result in a more balanced and healthy diet and lifestyle in general. We designed a randomized controlled trial to determine compliance with the Smarter Pregnancy intervention and investigate whether use of the program empowers women to improve nutrition prior to conception and during early pregnancy.

## Methods

### Trial Design, Participants, and Recruitment

A detailed study protocol is published elsewhere [34]. In short, women between aged 18 and 45 years were considered eligible for inclusion in this study if they were in possession of a smartphone with internet access, resided in the Netherlands, and were contemplating pregnancy or already pregnant (<13 weeks of pregnancy). We excluded women if they had insufficient knowledge or understanding of the Dutch language, if they were being treated by a dietician to lose weight in the context of fertility treatment, or if they were on a vegan diet. Body mass index (BMI) was not an exclusion criterion. Dutch-speaking male partners with smartphones were also invited to participate unless they were receiving dietary advice or were on a vegan diet.

We performed a single-center, open randomized controlled trial in the urban area of Rotterdam, the Netherlands. There was no blinding of participants, involved health care professionals, or involved researchers. Women eligible for inclusion were invited to participate by a health care professional working in one of the following locations in this area: one academic hospital, four teaching hospitals, four midwifery practices, and several children’s daycare and child health centers. After online registration, each woman was contacted by a researcher to verify her eligibility, provide her with more details about the study, answer any questions about the Smarter Pregnancy program, and confirm her inclusion in the study. After the researcher had verified the eligibility of women willing to participate in the study, inclusion occurred by signing an online (ie, digital) patient informed consent form, which was sent by email to the participant through a secure study email account. Participants were asked to print and sign the informed consent form to ensure compliance with the guidelines laid down in the Declaration of Helsinki. Participants were able to resign from the study at any time without having to give a reason. All procedures involving patients were approved by the medical ethical and institutional review board of the Erasmus Medical Center, University Medical Centre, Rotterdam, the Netherlands. Trial was registered at Netherlands Trial Register [NL3927].

### Randomization

Randomization was stratified according to the location from which the participants had been recruited. A preprogrammed permuted blocking design (two intervention and two control allocations per block) ensured that the number of women from the different locations was balanced between the two treatment groups and allocation into groups was concealed from the researchers. Men were always assigned the same group as their female partner.

### Intervention

The design and development of the lifestyle change intervention led to the availability of two versions that we could use in our study: a full version that included all functionality and personalized interaction (the intervention), and a modified version that had limited functionality and no personalized interaction, which was used in the control group. Detailed information on the program can be found in the study protocol [34]. Because the focus of this study was on evaluating the change in the intakes of vegetables and fruits, details on the intake of other food groups and cessation of smoking and alcohol consumption are not further addressed.

Men and women in the intervention group received tailored coaching based on their answers of the baseline questionnaire to questions regarding vegetable, fruit, and folic acid supplement intake. Vegetable intake and fruit intake were both subdivided into a risk score of 0, 1.5, or 3, where 0 represented an adequate daily intake (vegetable intake of ≥200 grams per day or a fruit intake of ≥2 pieces per day). A score of 1.5 represented a nearly adequate intake (vegetable intake of 150 to 200 grams per day or a fruit intake of 1.5 to 2 pieces per day). A score of 3 represented an inadequate daily intake (vegetable intake <150 grams per day or a fruit intake of <1.5 pieces per day). Folic acid supplement use was considered adequate (score 0) or inadequate (score 3) based on the international recommended dose of 400 μg per day. The dietary risk score (DRS) was calculated as the sum of the scores for vegetable, fruit, and folic acid supplement intake, thus ranging from 0 to 9 in women, in which 9 was the most unhealthy risk score. In men, the DRS ranged from 0 to 6, as they did not receive any coaching regarding folic acid supplement use.

The tailored coaching comprised a maximum of three emails or text messages per week. These emails and messages contained seasonal recipes, incentives, feedback, recommendations, and additional questions regarding the participant’s diet. Progress regarding the adoption of healthy behavior was monitored using online questionnaires at 6, 12, 18, and 24 weeks in the intervention group, while the control group only received these questionnaires at 12 and 24 weeks.

The first follow-up study questionnaire was sent at 36 weeks (ie, 12 weeks after the final screening questionnaire) and contained the same questions on nutrition, lifestyle, and pregnancy status as the other online questionnaires at baseline, 12, and 24 weeks.

All participants were given access to a personal online webpage that provided access to additional modules (ie, apps) that promoted physical activity, a calendar to improve compliance with hospital appointments and taking their folic acid supplements, and a module to monitor the safety of any prescribed medication.

### Outcome Measures

The main outcome measures of this study were compliance of all participants, defined as the percentage of participants who completed the online screening at 24 weeks, and degree of improvement in nutrition in women 24 weeks after starting the Smarter Pregnancy program, as reflected by a reduction in the DRS.

### Statistical Analysis

We analyzed data from all participants, those who completed the Smarter Pregnancy program and those who resigned prematurely, whereby missing data were handled using the last-observation-carried-forward method.

For all participants, the DRS was calculated at baseline (*t*=0), 12 weeks (*t*=12), and 24 (*t*=24) weeks. In further analyses, we included all women with a DRS>0 at baseline since these women were able to improve unhealthy behaviors and thereby reduce their risk scores. The primary analysis was based on intention to treat. The difference in differences principle was used to analyze the continuous outcome measures used in a multivariable linear regression model, adjusted for the baseline value of the DRS. Bootstrapping was performed on all analyses because residuals of the linear regression analyses were not normally distributed [35]. All analyses were performed using SPSS Statistics for Windows version 21.0 (IBM Corp). The analyzed dataset of this trial will be available from the corresponding author upon reasonable request.

### Patient Involvement

The design of this trial was based partly on patient evaluations obtained during a survey of the Smarter Pregnancy program [12]. During this study, we also received questions and feedback from participants, which we used to optimize trial procedures and improve participant satisfaction.

## Results

### Participant Characteristics

**Figure 1:**
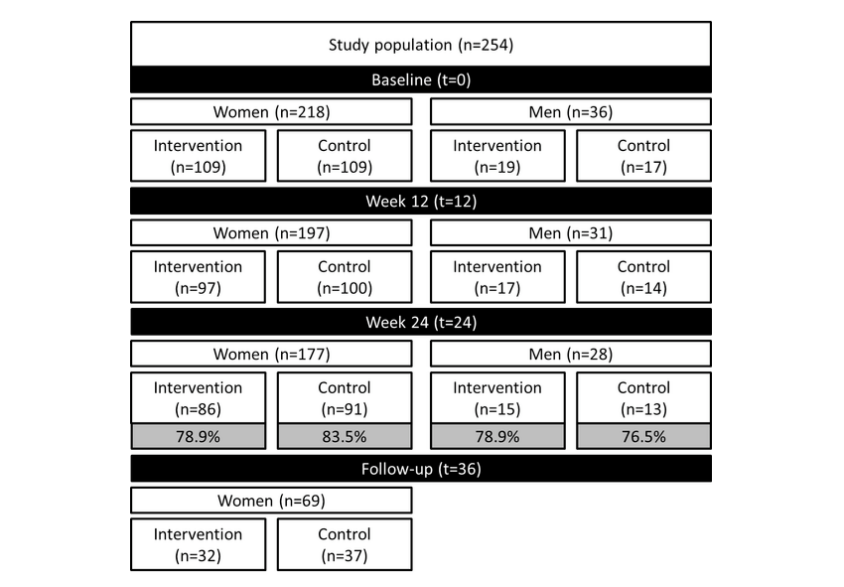
Flowchart of the study population stratified by sex.

The study was open from May 2014 until January 2017 and included 218 women and 36 men. After randomization, the intervention group consisted of 109 women and 19 men and the control group of 109 women and 17 men (Figure 1). Baseline characteristics of all women in the study population are shown in Table 1. Median age, median BMI, pregnancy status, and partner participation were similar for women in the intervention and control groups. In our population, the BMI of all participants ranged between 16.3 to 45.9 kg/m^2^. In both groups, most women were highly educated and of Dutch origin.

Regarding nutrition and lifestyle, in both groups almost two-thirds of women reported an inadequate vegetable intake. Fruit intake was inadequate in about one-third of women in both groups. In both groups, almost 1 in 10 women reported inadequate folic acid supplement use. These figures resulted in a median DRS at baseline of 3 in both groups. The baseline characteristics of the participating men are shown in Multimedia Appendix 1; in both groups, men had a higher DRS and a higher prevalence of smoking compared with women.

**Table 1:** Baseline characteristics of all women in the intervention and control groups.

Characteristics		Intervention n=109	Control n=109
Age in years, median (IQR^a^)		30.6 (5.3)	30.7 (5.7)
Height (cm), median (IQR)		170.0 (9)	170.0 (9)
BMI^b^ (kg/m^2^), median (IQR)		24.2 (6.0)	23.7 (5.4)
Pregnant at enrollment, n (%)		36 (33.0)	37 (33.9)
Partner participation, n (%)		19 (17.4)	18 (16.5)
**Geographic origin, n (%)**			
	Dutch	83 (76.1)	86 (78.9)
	Western	5 (4.6)	2 (1.8)
	Nonwestern	15 (13.8)	15 (13.8)
	Missing	6 (5.5)	6 (5.5)
**Education, n (%)**			
	High	62 (56.9)	76 (69.7)
	Intermediate	37 (33.9)	23 (21.1)
	Low	1 (0.9)	3 (2.8)
	Missing	9 (8.3)	7 (6.4)
**Vegetables, grams per day, n (%)**			
	<150 (DRS^c^ 3)	65 (59.6)	65 (59.6)
	150-200 (DRS 1.5)	19 (17.4)	24 (22.0)
	≥200 (DRS 0)	25 (22.9)	20 (18.3)
**Fruit, pieces per day, n (%)**			
	<1.5 (DRS 3)	39 (35.8)	37 (33.9)
	1.5-2.0 (DRS 1.5)	8 (7.3)	14 (12.8)
	≥2.0 (DRS 0)	62 (56.9)	58 (53.2)
**Folic acid supplement use, n (%)**			
	Inadequate (DRS 3)	10 (9.2)	10 (9.2)
	Adequate (DRS 0)	99 (90.8)	99 (90.8)
DRS, median (IQR) (DRS 0-9)		3 (4.5)	3 (3.0)
Alcohol consumption, n (%)		85 (78.0)	82 (75.2)
Smoking, n (%)		5 (4.6)	12 (11.0)

^a^IQR: interquartile range.

^b^BMI: body mass index.

^c^DRS: dietary risk score.

### Compliance and Dropout

Of women entering the study, compliance was 81.2% (177/208). In the intervention group, compliance was 78.9% (86/109) and in the control group 83.5% (91/109; *P*=.95). In men, overall compliance was 77.8% (28/36), with 78.9% (15/19) in the intervention group and 76.5% (13/17) in the control group (*P*=.59). When we compared the baseline characteristics of all women who completed the program (n=177) with those of women who resigned prematurely (n=41), we observed that women who resigned prematurely had a significantly higher median DRS (*P=*.007) and a significantly lower level of education (*P*=.01; Table 2).

**Table 2:** Baseline characteristics of all women, stratified by compliance, defined as whether they completed the questionnaire at 24 weeks or resigned before this time point.

Characteristics		Completed n=177	Resigned n=41	*P* value
Age in years, median (IQR^a^)		30.8 (6.0)	30.1 (6.0)	.33
Height (cm), median (IQR)		170 (10.0)	169 (8.0)	.86
BMI^b^ (kg/m^2^), median (IQR)		23.4 (5.8)	25.6 (4.5)	.13
Pregnant at enrollment, n (%)		63 (33.9)	10 (31.3)	.77
Partner participation, n (%)		31 (16.7)	6 (18.8)	.77
**Geographic origin, n (%)**				.21
	Dutch	145 (78.0)	24 (75.0)	
	Western	7 (3.8)	0 (0)	
	Nonwestern	27 (14.5)	3 (9.4)	
	Missing	7 (4.0)	5 (12.2)	
**Education, n (%)**				.01
	High	119 (64.0)	19 (59.4)	
	Intermediate	53 (28.5)	7 (21.9)	
	Low	3 (1.6)	1 (3.1)	
	Missing	11 (6.2)	5 (12.2)	
**Vegetables, grams per day, n (%)**				.13
	<150 (DRS^c^ 3)	106 (57.0)	24 (75.0)	
	150-200 (DRS 1.5)	38 (20.4)	5 (15.6)	
	≥200 (DRS 0)	42 (22.6)	3 (9.4)	
**Fruit, pieces per day, n (%)**				.06
	<1.5 (DRS 3)	59 (31.7)	17 (53.1)	
	1.5-2.0 (DRS 1.5)	19 (10.2)	3 (9.4)	
	≥2.0 (DRS 0)	108 (58.0)	12 (37.5)	
**Folic acid supplement use, n (%)**				.48
	Inadequate (DRS 3)	16 (8.6)	4 (12.5)	
	Adequate (DRS 0)	170 (91.4)	28 (87.5)	
DRS, median (IQR) (DRS 0-9)		3 (4.5)	4.5 (3.0)	.007
Alcohol consumption, n (%)		40 (21.5)	11 (34.4)	.14
Smoking, n (%)		13 (7.0)	4 (12.5)	.28

^a^IQR: interquartile range.

^b^BMI: body mass index.

^c^DRS: dietary risk score.

### Dietary Risk Score

**Figure 2:**
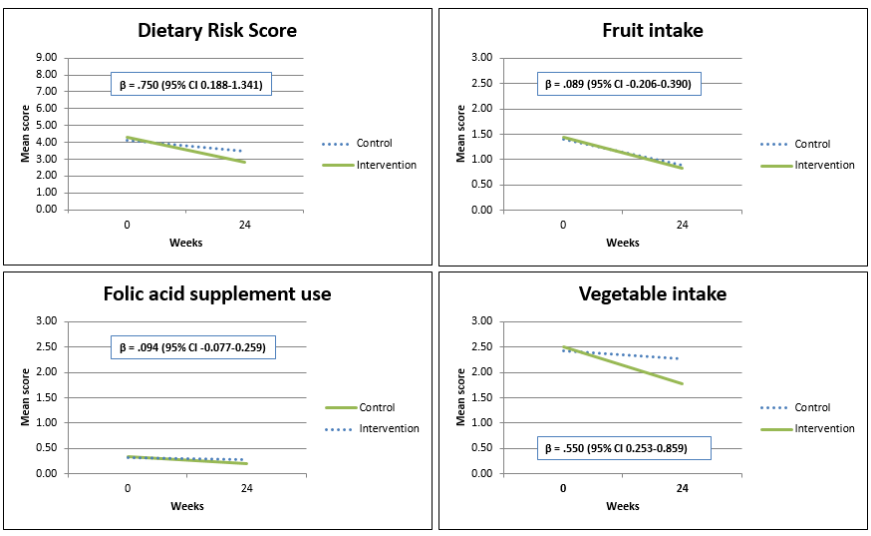
Dietary risk score (DRS) and separate risk factors over time in all women (with a baseline DRS>0) in the intervention and control groups. The linear regression model includes adjustment for baseline DRS and randomization.

The outcomes of the multivariable linear regression model regarding the DRS and separate risk factors are depicted in Figure 2. Compared with participants in the control group, participants in the intervention group showed a significantly larger reduction in the DRS (*β*=.750; 95% CI 0.188-1.341), in particular for vegetable intake (*β*=.550; 95% CI 0.253-0.859). There were no significant differences between groups regarding fruit intake and folic acid supplement intake.

### Follow-Up

**Figure 3:**
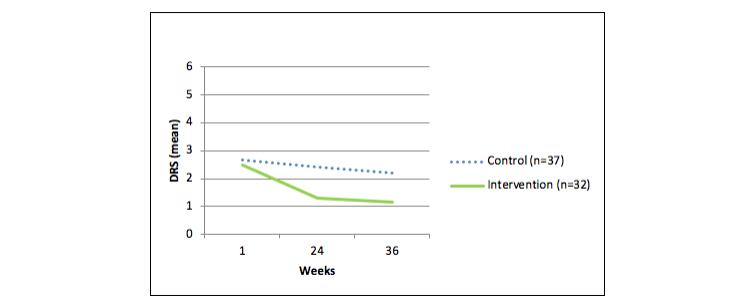
Mean dietary risk score (DRS) over time in all women who completed the follow-up questionnaire at 36 weeks (n=69).

A total of 69 women (27.1%) completed the follow-up questionnaire 36 weeks after randomization. Although women did not receive any coaching during the period between 24 and 36 weeks, the mean DRS appeared to continue to decrease in both the intervention group (n=32) and control group (n=37; Figure 3). All men were lost to follow-up regarding the questionnaire at 36 weeks.

## Discussion

### Principal Findings

The results of this randomized controlled trial indicate that compliance with the empowering lifestyle change intervention Smarter Pregnancy is high. Our findings also demonstrated that the use of this program improved the intake of vegetables (in particular) in women prior to conception and during early pregnancy. The follow-up results also suggest that this intervention had a positive effect on the continuation of healthier nutrition intake 12 weeks after stopping the intervention.

### Strengths and Limitations

Major strengths of this study are the randomized controlled trial design, the fact that we used a standard and light version of the Smarter Pregnancy intervention, and the uniform collection of data in both the intervention and control group at baseline, 12, 24, and 36 weeks. By providing the control group with limited information and interaction, we encouraged participants in this group to adhere to the program, thereby ensuring high compliance and preventing dropout. The high compliance rates observed in both groups support this strategy. A further strength is that a wide range of professionals and non–health care professionals recruited women for the study, as women were approached not only during a scheduled hospital or midwifery visit, but also at children’s day care centers, for example. Women who had not been personally invited could also enroll via the website we set up, thereby limiting selection bias. Additional strengths of this study are the longitudinal observations, the fact that male partners also participated, and that we collected additional information regarding lifestyle factors, educational level, geographic origin, and pregnancy status at enrollment.

In terms of weaknesses, we experienced difficulties enrolling a sufficient amount of women in the preconception period, which was the reason we expanded our inclusion criteria to include women up to 13 weeks of pregnancy. This meant that our sample size was limited, which prevented us from carrying out subgroup analyses that would have provided additional quantitative data regarding lifestyle, fertility, pregnancy course and outcome, and cost effectiveness.

We decided to reduce the total number of questionnaires in the control group from 4 to 2 because we expected that further reduction of the number of questionnaires to 1, or even 0, would have led to a higher number of resigning participants. This led to a difference in questionnaire administration, which can be considered a limitation.

A further limitation was that the program Smarter Pregnancy was only available online and in the Dutch language, thereby excluding women who have insufficient knowledge of this language or no internet access from participation in this trial. This might have excluded a high-risk population of women who might have benefitted the most from the program. An English version of this program will be released very soon.

### Comparison With Other Studies

To date, there is little scientific evidence for the success of nutrition and lifestyle interventions during the preconception or periconception period. Most studies regarding preconception interventions have focused on micronutrient supplementation or weight gain, for example [36,37], or on specific subgroups and disease-related conditions, such as fertility treatment [38], polycystic ovary syndrome [39], or pre-existing/gestational diabetes [40,41]. Even fewer studies have looked at interventions on the mobile phone specifically focused on the preconception and periconception periods [40,42].

The small amount of scientific evidence regarding mHealth during the periconception period mainly showed comparable results regarding the prevalence of unhealthy nutrition and lifestyle, especially insufficient fruit and vegetable intake and smoking. These and other studies also showed and specifically addressed the relatively high dropout rates among all users [43-46]. There is a clear need for high-quality evidence that intervening in these periods in general is indeed effective, since many studies could also not demonstrate significant effectiveness [45,47,48]. A key problem underlying this lack of evidence is the lack of awareness of the importance of periconception care among both patients and health care professionals, resulting in low adherence and uptake of such care. This was described in 2002 by De Weerd et al [49]. While this barrier is widely acknowledged, and various studies have focused on how to overcome it, unfortunately barriers still remain [24,28,50,51]. It has been suggested that modern marketing campaigns such as those increasingly found online might help to overcome or at least lower this barrier [30]. Taking this into account, together with the wide uptake of mobile devices and online information [52], we believe that our approach—using a personalized intervention on the mobile phone specifically targeted at identifying and improving periconceptional risk factors—can contribute to lowering the unawareness barrier.

### Conclusions and Future Perspectives

To our knowledge, Smarter Pregnancy is the first intervention on the mobile phone showing effectiveness in empowering women to improving healthy nutrition before and during early pregnancy. We therefore consider this study a good example of a successful intervention study, of which the findings support the considerable potential of using mobile phone apps. Current awareness among health care professionals of their responsibility to inform their patients about healthy nutrition is very low [53]. However, we assume that the increasing amount of evidence for the importance of nutrition in the periconception period will make health care professionals particularly more aware and make them more likely to recommend evidence-based interventions to their patients. This will contribute to an increase in the general awareness of the importance of the periconception period. As a result, we hope that periconception care will become more easily and more widely accessible, thereby improving reproductive and pregnancy outcomes in both fertile and subfertile couples.

## Appendix

Multimedia Appendix 1 Baseline characteristics of all men in the intervention and control groups.

Multimedia Appendix 2 CONSORT-eHEALTH 2010 checklist.

## Abbreviations

BMI: body mass index
DRS: dietary risk score
eHealth: electronic health
mHealth: mobile health
NCD: noncommunicable disease
ZonMW: Netherlands Organization for Health Research and Development
