# Supplementary material for: A Mobile App Lifestyle Intervention to Improve Healthy Nutrition in Women Before and During Early Pregnancy: Single-Center Randomized Controlled Trial
Source: J Med Internet Res. 2020 May 15;22(5):e15773. doi: 10.2196/15773 (PMC7260659; doi:10.2196/15773)
Supplement: Multimedia Appendix 1 [file jmir_v22i5e15773_app1.docx]

**Supplementary Table 1:** Baseline characteristics of all men in the intervention and control groups.

| **Men (n=36)** | | **Intervention**  **(n=19)** |  | **Control**  **(n=17)** |  |
| --- | --- | --- | --- | --- | --- |
|  | |  | **Missing** |  | **Missing** |
| **Age (years), median (IQR)** | | 31.7 (5.0) |  | 32.0 (4.6) |  |
| **Height (cm), median (IQR)** | | 182 (11) |  | 183 (10) |  |
| **BMI (kg/m^2^), median (IQR)** | | 24.3 (6.4) |  | 25.8 (3.5) |  |
|  | |  |  |  |  |
| **Partner pregnant at enrolment, n (%)** | | 2 (10.5) |  | 1 (5.9) |  |
|  | |  |  |  |  |
| **Geographic origin** | |  | 1 |  | 5 |
| Dutch, n (%) | | 13 (68.4) |  | 11 (64.7) |  |
| Wwestern, n (%) | | 1 (5.3) |  | 0 |  |
| Non-western, n (%) | | 4 (21.1) |  | 1 (5.9) |  |
| **Education** | |  | 1 |  | 5 |
| High, n (%) | | 12 (63.2) |  | 10 (58.8) |  |
| Intermediate, n (%) | | 5 (26.3) |  | 2 (11.8) |  |
| Low, n (%) | | 1 (5.3) |  | 0 |  |
|  | | | | | |
| **Vegetables, grams per day** | **DRS** |  | 0 |  | 0 |
| <150, n (%) | 3 | 8 (42.1) |  | 9 (52.9) |  |
| 150-200, n (%) | 1.5 | 6 (31.2) |  | 3 (17.6) |  |
| ≥200, n (%) | 0 | 5 (26.3) |  | 5 (29.4) |  |
| **Fruit, pieces per day** |  |  | 0 |  | 0 |
| <1.5, n (%) | 3 | 8 (42.1) |  | 6 (35.3) |  |
| 1.5-2.0, n (%) | 1.5 | 2 (10.5) |  | 3 (17.6) |  |
| ≥2.0, n (%) | 0 | 9 (47.4) |  | 8 (47.1) |  |
| **DRS, median (IQR)** | 0-6 | 3 (3) |  | 3 (4.5) |  |
|  |  |  |  |  |  |
| **Alcohol consumption, n (%)** | - | 15 (78.9) |  | 11 (64.7) |  |
| **Smoking, n (%)** | - | 5 (26.3) |  | 3 (17.6) |  |

DRS: dietary risk score, BMI: body mass index, IQR: interquartile range
